# Developmental exposure to corn grown on Lake Erie dredged material: a preliminary analysis

**DOI:** 10.3389/fnbeh.2023.987239

**Published:** 2023-04-21

**Authors:** Kaylyn A. S. Flanigan, Madelyn I. Czuba, Victoria R. Riesgo, Megan A. Rúa, Louise M. Stevenson, Jari Willing

**Affiliations:** ^1^J.P. Scott Center for Neuroscience, Mind, and Behavior, Department of Psychology, Bowling Green State University, Bowling Green, OH, United States; ^2^Department of Biological Sciences, Wright State University, Dayton, OH, United States; ^3^Department of Biological Sciences, Bowling Green State University, Bowling Green, OH, United States; ^4^Oak Ridge National Laboratory, Environmental Sciences Division, Oak Ridge, TN, United States

**Keywords:** soil amendment, neurotoxicity, neurodevelopment, open field, object recognition

## Abstract

While corn is considered to be a healthy food option, common agricultural practices, such as the application of soil amendments, might be introducing contaminants of concern (COC) into corn plants. The use of dredged material, which contain contaminants such as heavy metals, polychlorinated biphenyls (PCBs) and polycyclic aromatic hydrocarbons (PAHs), as a soil amendment is increasing. Contaminants from these amendments can accumulate in corn kernels harvested from plants grown on these sediments and potentially biomagnify in organisms that consume them. The extent to which secondary exposure to such contaminants in corn affect the mammalian central nervous system has been virtually unexplored. In this preliminary study, we examine the effects of exposure to corn grown in dredge amended soil or a commercially available feed corn on behavior and hippocampal volume in male and female rats. Perinatal exposure to dredge-amended corn altered behavior in the open-field and object recognition tasks in adulthood. Additionally, dredge-amended corn led to a reduction in hippocampal volume in male but not female adult rats. These results suggest the need for future studies examining how dredge-amended crops and/or commercially available feed corn may be exposing animals to COC that can alter neurodevelopment in a sex-specific manner. This future work will provide insight into the potential long-term consequences of soil amendment practices on the brain and behavior.

## 1. Introduction

The United States is the greatest producer and consumer of corn in the world (Gloy, 2017; McConnell, 2021). Additionally, corn is a major source of carbohydrates for humans and livestock and recommended as part of a balanced diet (satisfying vegetable and fiber requirements) by governmental and non-governmental authorities (U.S. Department of Health and Human Services, and U.S. Department of Agriculture, 2015; U.S. Department of Agriculture, and U.S. Department of Health and Human Services, 2020; McConnell, 2021); however, corn may contain contaminants of concern (COC), naturally occurring compounds that are typically regulated due to potentially toxic effects, acquired from the soil on which it is grown. This may be especially true for corn grown on soils amended with dredged sediments that are increasingly sourced from industrial areas. For example, sediments dredged from Lake Erie shipping lanes have been proposed as a soil amendment in Ohio due to their ability to mitigate nutrient depletion as it is rich in nutrients, organic matter and has high water retention (Bhairappanavar et al., 2018). Dredged sediments have also been shown to contain COC including heavy metals, polychlorinated biphenyls, polycyclic aromatic hydrocarbons, pharmaceuticals and other emerging COC, chemicals or toxicants that may have an impact on human health but are not yet regulated (Hull and Associates Inc, 2018). These contaminants persist and accumulate within the environment and can potentially biomagnify in animals who consume plants grown on these sediments (Darmody et al., 2004; Ebbs et al., 2006). The COC within dredged sediments poses particular concern for the developing brain since early developmental periods are characterized by heightened susceptibility to toxicants. Because dredged material in Ohio will likely be applied to fields growing corn for animal consumption, commercially available feed corn was used as a reference comparison to maintain biological relevance. While this feed corn may not be entirely devoid of COC, future studies will assess the effects of corn from a variety of sources in comparison to a group with no corn exposure.

Gestational and perinatal exposure to many COC found in dredged material may affect neurodevelopmental processes such as neurogenesis, gliogenesis, synaptogenesis, apoptosis, and migration, and can alter a variety of behaviors (Bushnell et al., 2002; Naveau et al., 2014; Hossain et al., 2016; Rao Barkur and Bairy, 2016). Numerous studies suggest that the developing perinatal brain is more susceptible to the effects of COC, as these toxicants can cross the placenta and interfere with the developmental processes mentioned above (Knudsen, 2004). One region that is particularly sensitive to environmental toxins is the hippocampus (Stoltenburgdidinger, 1994; Reddy et al., 2007; Pierozan et al., 2020). Exposure to COC during gestation and lactation can impair the growth of this region and can cause long-term changes in hippocampal-dependent behaviors involving learning and memory, sometimes in a sex-specific manner (Curtis et al., 2011; Karlsson et al., 2015). Because corn grown on dredged material is likely to contain numerous bioaccumulated COC that alter neurodevelopment and behavior in isolation, the current preliminary study examines the effects of pre- and postnatal exposure to this corn and/or commercial feed corn on the neural, physical, and behavioral development of male and female Long-Evans rats.

## 2. Methods

### 2.1. Subjects

Adult male and female Long Evans rats (approximately 3–4 months of age) were purchased from Envigo (Indianapolis, IN, USA) to be used as breeders. The offspring of these breeders were used as experimental subjects. All animals were kept on a 12:12 h light/dark cycle with lights off at 20:00 and were housed in polycarbonate cages with corn cob bedding and glass water bottles. Animals were weaned on postnatal day (P) 25 and were housed in pairs or triplets with same-sex littermates. Throughout the study, animals had *ad libitum* access to food and water. The diet utilized was the Teklad Rodent Diet 8604 (Envigo), which is primarily composed of soybeans, wheat and corn. Experimental corn exposure (described below) was a supplement to this *ad libitum* diet. All experimental procedures were approved by Bowling Green State University’s (BGSU) Institutional Animal Care and Use Committee (IACUC, protocol: 1485417) prior to the study.

### 2.2. Growth of dredge-amended corn and COC profiles

Corn (*Zea mays*) was grown on dredged sediments at the Great Lakes Dredged Material Center for Innovation (GLDMCI) located in Toledo, Ohio (41.6700354oN–83.5029711oW). Sediments were originally dredged from Toledo Harbor and the Maumee River in 2016–17 and allowed to dewater between 2017 and 18 before being planted with corn (variety: W2903DP; Wellman Seeds Inc., Delphos, OH, USA) during the 2019 growing season (Rúa et al., 2023). Existing vegetation was sowed into the dredged material prior to sowing rows of corn. All plots were sprayed with a commonly used herbicide mixture containing glycophosate (1.5 qt/acre, 53.8%) and ammonium sulphate (1.5 lb/acre, 48%) in 20 gallons of water per acre (Rúa et al., 2023). This herbicide mixture is standard practice and is not unique to corn growth with dredge amendments. Ears of corn were hand harvested at the end of the growing season and then dried in a drying oven at 55°C for at least 48 h and then stored with desiccant until use. Analysis of COC profiles of commercial feed and dredge-amended corn is ongoing as part of a larger collaboration.

### 2.3. Corn dosing

Animals were randomly assigned to receive corn grown on dredged material or commercially available feed corn grown without the use of soil amendments (Tractor Supply Inc). Whole corn kernels were presented as a dietary supplement (without food restriction) at a dose of 6 g/kg. One cohort of subjects received this exposure in adulthood. These animals were given either type of corn for 20 consecutive days from P120—P139 (5 males and 4 females in each corn group: Adult Exposure Group). This preliminary study was included to investigate the potential for acute toxic effects in adults prior to administering this corn to pregnant dams. As these data are preliminary and since no sex differences were anticipated, this smaller cohort of animals was utilized for the adult exposure groups. In a second cohort (12 males and 11 females in each corn group: Developmental Exposure Group), corn exposure took place during gestation and lactation. Dams were given either corn type from embryonic day 0 throughout gestation and lactation until the day of weaning. All animals rapidly consumed all the corn within several minutes. The offspring of these exposed dams were used to assess behavior and neuroanatomy in adulthood. Eleven females and 12 males were developmentally exposed to commercial feed corn, while 11 females and 12 males were exposed to dredge-amended corn. There was no effect of corn exposure on litter size, pup sex ratio or body weight (data not shown).

### 2.4. Behavior testing

Behavioral tests were conducted in 9ft. × 9ft. temperature-controlled room within the vivarium. All animals, regardless of testing day, were acclimated in their home cages to the testing room for at least 5 min, but no longer than 15 min, before being tested. All arenas were cleaned with 30% ethanol and allowed to dry between subjects. All tests were scored by experimenters blind to corn exposure during behavior testing.

Subjects exposed to the corn as adults were behaviorally tested in the Elevated Plus Maze (EPM) 1 day after their last dose of corn. The EPM is composed of two perpendicular arms, one arm being walled or enclosed and the other being open and is used to measure anxiety-like behavior (Lister, 1987). For a period of 5 min, experimenters manually assessed the amount of time spent in the open arms, the latency to enter an open arm, the number of entries into open arms and the number of total arm entries. Animals in the developmental study were weaned on P25 and began behavior testing on P70 with the EPM. EPM behavior was administered first to assess baseline anxiety-like behavior prior to further behavior testing and maze/apparatus exposure. The following day, animals were tested in the Open Field task (OF). The OF arena was a 70 cm × 70 cm plexiglass box with walls 40 cm high and a 15 cm × 15 cm “center area.” Animals were allowed to explore the box freely for a 5 min period while the experimenter recorded the latency to enter the center, the amount of entries into the center and the amount of time in the center. Center activity in the OF has also been associated with anxiety-like behavior (Zohner, 1968; Tartar et al., 2009).

The next day, animals were tested in the Novel Object Recognition task (NOR), which examines exploratory behavior toward novel objects, and recognition memory (Ennaceur and Delacour, 1988; Ennaceur and Meliani, 1992; Lueptow, 2017). This test consisted of two trials. In the first, the animal is placed in the OF test box with two identical objects equidistant from two corners of the box. Animals were allowed to freely explore the objects for a period of 5 min, and the experimenter recorded interaction time with each object. After a 2 h inter-trial interval, there is a second trial where one of the identical objects is replaced with a novel object, and the amount of interaction time with each object is recorded. More time with the novel object is considered to indicate greater recognition memory, as rodents typically choose to interact with novel elements in their environment. Objects used in the NOR test were of similar size and weight, used previously with no inherent preference indicated, and cleaned between subjects. These three behavioral tests were included in this preliminary experiment as they are standard tests used to assess exploratory behavior, anxiety and recognition memory in laboratory subjects. Future work will employ a more comprehensive battery of behavior tests to assess other facets of cognition.

### 2.5. Histology—assessment hippocampal volume

Following behavior testing, a subset of subjects (*n* = 4 males and 3 females per feeding group; total *n* = 14) were anesthetized with a lethal dose of sodium pentobarbital and perfused with 4% paraformaldehyde. After brain extraction, tissue was stored in 4% paraformaldehyde for 48 h and then moved to a 30% sucrose solution for 72 h, after which it was sliced. Fixed tissue was cut into 40-micron with a freezing microtome and sections every 4^th^ section containing the hippocampus was mounted on glass slides. Sections were stained with methylene blue-azure II as previously described (Markham et al., 2006; Willing and Juraska, 2015). Volumetric measurements were gathered using MicroBrightField Biosciences Stereoinvestigator software. Using a 2.5X objective, the hippocampus (including all layers/subregions), beginning at −1.80 mm to −5.60 from bregma, was traced on each section for each subject (approximately 25 sections per subject). Hippocampal areas were summed together to reach a combined area per subject, then multiplied by 40 microns, a thickness constant, resulting in an estimate of hippocampal volume. A subset of behavioral subjects was chosen to investigate the possibility of neuroanatomical changes between commercial corn and dredge corn groups.

### 2.6. Statistical analyses

For both Adult Exposure and Developmental Exposure experiments, behavioral measures of the EPM, OF, and NOR were compared using two-way ANOVA (Corn type × Sex) followed by Tukey HSD *post-hoc* tests. For the NOR test, an additional analysis for total object exploration was conducted using Students *t*-tests (conducted separately for males and females). Hippocampal volume was analyzed using a two-way ANOVA (Corn type × Sex). All statistical tests were conducted using SPSS Version 27.0.0.0 and *p*-values were determined to be significant when *p* < 0.05.

## 3. Results

### 3.1. Adult exposure

There was no main effect of corn treatment on any measure in the EPM [closed entries, *F*(1,14) = 0.00705, *p* = 0.934; open entries, *F*(1,14) = 1.35, *p* = 0.264; end visits, *F*(1,14) = 0.422, *p* = 0.562; open arm time, *F*(1,14) = 2.08, *p* = 0.171; open latency, *F*(1,14) = 1.16, *p* = 0.300; data not shown). There was a main effect of sex on open arm time [open arm time, *F*(1,14) = 4.96, *p* = 0.043; data not shown] but not for any other measure of the EPM. This effect was driven by dredge-amended corn females that exhibited increased time spent in the open arms. There were no significant differences in body weight [*F*(1,14) = 0.00944, *p* = 0.924] or brain weight [*F*(1,14) = 0.0214, *p* = 0.886] observed between corn treatments.

### 3.2. Developmental exposure

#### 3.2.1. Behavior

**FIGURE 1:**
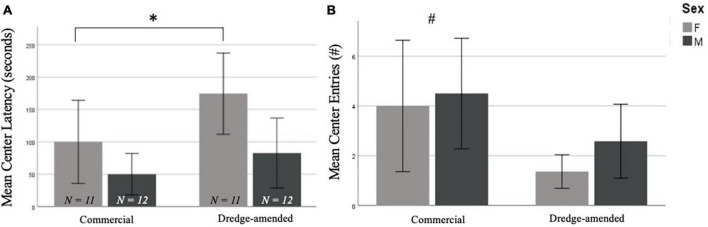
Early exposure to dredge-amended corn affects adult Open Field Test (Total *N* = 46; *N* = 12 males and 11 females per corn group). Mean center latency in seconds **(A)** and mean number of center entries **(B)** in the Open Field Test with standard error bars. Females in both commercial and dredge-amended groups exhibited increased center latencies compared to males in either group (sex-specific difference indicated by *). A non-significant trend showed increased latencies in the dredge-amended group. A corn treatment difference (indicated by #) was observed for the number of center entries. Dredge-amended groups had significantly fewer center entries compared to the commercial feed corn group.

There were no significant effects of corn type on performance in the EPM [closed entries, *F*(1,42) = 1.518, *p* = 0.23, open entries, *F*(1,42) = 0.863, *p* = 0.36, end visits, *F*(1,42) = 0.701, *p* = 0.41, open arm time, *F*(1,42) = 0.627, *p* = 0.43, open latency, *F*(1,42) = 0.141, *p* = 0.71; data not shown]. In the OF test, there was no difference between groups for time spent in the center of the arena [*F*(1,42) = 1.824, *p* = 0.18], but there was a significant difference in the number of center entries by corn treatment [*F*(1,42) = 5.672, *p* = 0.02; Figure 1B]. The commercial feed corn group exhibited more center entries than the dredge-amended group; this effect was most prominent in dredge-amended females, who had the lowest average of center entries. There was also a significant difference in center latency between males and females [*F*(1,42) = 6.873, *p* = 0.012; Figure 1A] with females exhibiting increased latencies. A non-significant trend was also found between corn treatment and center latency [*F*(1,42) = 3.933, *p* = 0.054, Figure 1A], with subjects exposed to dredge-amended corn having a longer latency. There was not a significant interaction between sex and corn treatment for center latency [*F*(1,42) = 0.595, *p* = 0.45].

**FIGURE 2:**
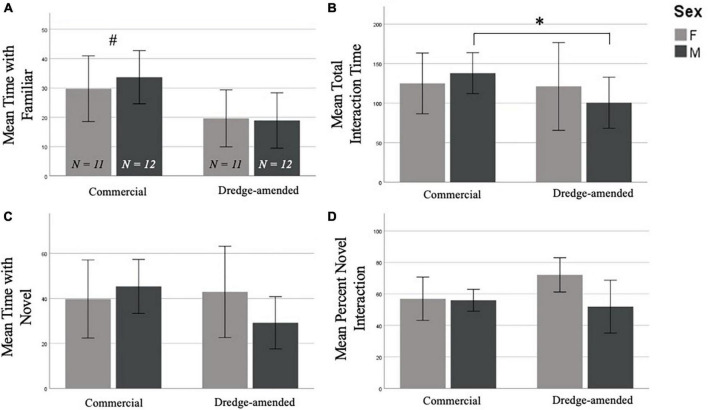
Early exposure to dredge-amended corn affects adult Novel Object Recognition Test (Total *N* = 46; *N* = 12 males and 11 females per corn group). Mean time spent with the familiar object **(A)**, mean combined trial 1 and trial 2 object interaction time **(B)**, mean time spent with the novel object **(C)**, and mean percent novel interaction **(D)**, percent novel interaction (Amount of Time with Novel/Total Object Interaction Time) of the Novel Object Recognition Test with standard error bars. There was an effect of corn treatment (indicated by #) on interaction with the familiar object; additionally, and dredge-amended males spent significantly less time interacting with objects compared to commercial feed corn males (indicated by *). Time spent with the novel object and percent novel measurements did not result in significant differences between corn treatment or sex.

In the NOR test there was a significant effect when examining interaction time with the familiar object compared to the novel object between corn treatments. Subjects in the commercial feed corn treatment spent significantly more time with the familiar object than subjects in the dredge amended corn treatment [*F*(1,42) = 6.354, *p* = 0.016; Figure 2A]. However, an analysis of total object exploration time (combining interaction time from trials 1 and 2) revealed that compared to subjects fed commercial feed corn, exposure to dredge-amended corn reduced total object interaction (novel and old objects, combined) in males (*t* = 2.375, *p* = 0.027; Figure 2B), but not females (*t* = 0.122, *p* = 0.904). There was no effect of corn type on the time (Figure 2C) or the percentage of time interacting with the novel object (F1,42 = 0.761, *p* = 0.388; Figure 2D; Percent Novel = Amount of Time with Novel / Total Object Interaction Time).

#### 3.2.2. Hippocampal volume

**FIGURE 3:**
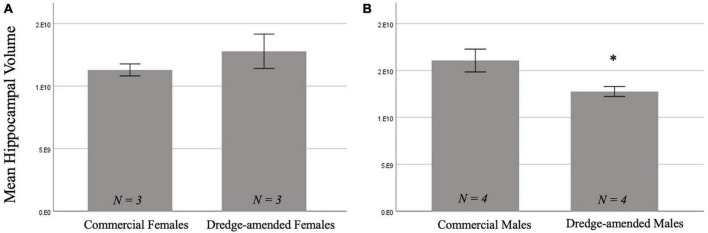
Early exposure to dredge-amended corn affects adult hippocampal volume. Mean hippocampal volume measurements for commercial and dredge-amended females [**(A)**; *N* = 6] and males [**(B)**; *N* = 8] with standard error bars. Dredge-amended males had a decrease (indicated by *) in hippocampal volume compared to their commercial counterparts. There was no significant effect of corn treatment in females.

There was not a significant main effect of corn type on hippocampal volume [*F*(1,10) = 3.325, *p* = 0.098]. There was a significant effect of sex [*F*(1,10) = 22.84, *p* < 0.001; Figure 3] with males having a greater hippocampal volume than females. Additionally, there was a significant interaction between corn treatment and sex [*F*(1,10) = 23.29, *p* < 0.001]. A *post-hoc* test revealed that developmental exposure to dredge-amended corn reduced hippocampal volume in adult male, but not female, rats compared to rats fed commercial feed corn (*p*_tukey_ = 0.002; Figure 3B).

## 4. Discussion

The purpose of the present study was to begin to examine potential long-term effects of corn grown on dredged sediments on mammalian behavior and neurodevelopment. Though the present study is preliminary, these results suggest that a dietary supplement of dredge-amended corn during an early critical period of brain maturation can affect both behavior and neuroanatomy compared to diets supplemented with commercially available feed corn. While adult exposure to dredge-amended corn in adulthood did not affect body weight, brain weight or behavior in the EPM, exposure during early development did have several significant effects. Developmental exposure to dredge-amended corn led to fewer entries into the center area of the OF, a slightly increased latency to enter the center, and a sex-specific decrease in time spent interacting with objects in the NOR. Additionally, developmental exposure to dredge-amended corn led to a sex-specific decrease in adult hippocampal volume.

Subjects from the developmental exposure study reflect a population that is more susceptible to COC influence. Toxicant exposure during critical periods of development can permanently alter brain development and behavior. Gestational and lactational exposure to corn grown on dredged sediment and commercially available feed corn allows for the examination of potentially indirect exposure to COC. The EPM and OF tests were utilized in the developmental exposure study to measure anxiety-like behavior. The OF test revealed that exposure to dredge-amended corn led to an increase in the number of center entries compared to commercial feed corn. While there were no significant findings from the EPM test, decreased center entries in an OF test does suggest a modest effect on anxiety-like or exploratory behavior induced by dredge-amended corn. Aside from anxiety, dredge-amended corn could also be affecting motivation and/or exploratory behavior. Interestingly, females of the dredge-amended corn group had the lowest average of center entries of all groups, suggesting that potential effects of dredge on anxiety-like behavior may reflect a heightened susceptibility in females. Results from the NOR test revealed that the commercial feed corn group had more interaction time with familiar old object in the second trial of the test. Though no effects were seen in the percentage of time spent with the novel object, this finding could suggest that commercial feed corn subjects had decreased recall of the old object, or that they experienced increased averse-to-novelty behaviors. However, this effect might have been driven in part by the fact that males exposed to dredge-amended corn spent less time overall interacting with objects in trials 1 and 2.

Developmental exposure to dredge-amended corn also reduced adult hippocampal volume, but only in male subjects. Previous studies have reported sex-specific effects following early environmental toxicant exposure. As complete contaminant profiles of dredge and commercial corn have not yet been determined; this preliminary study relies on previous work suggesting broad toxicant mixtures in dredge material (Hull and Associates Inc, 2018). These toxicant mixtures have been shown to induce sex-specific effects on the brain and behavior. For example, researchers dosed pregnant mice with either a single endocrine disrupting chemical (EDC) or a mixture. Results indicate that male progeny exposed to the mixture displayed a sex-specific behavior reversal in exploration time during the novel object recognition task (Sobolewski et al., 2014). Although an exposure to a single EDC or the mixture resulted in behavioral differences, this sex-specific reversal may suggest mixtures of EDC could impact male and female brains disproportionately. A similar study found that, following prenatal exposure to an EDC mixture delayed puberty in males but not in females (Gore et al., 2022). Since studying toxicant mixtures is often more clinically relevant given the mechanism of human exposure, most research in neurotoxicology focuses on assessing a single toxin in order to better determine a mechanism of action (Cory-Slechta, 2005).

Previous work suggests that the hippocampus is a target for a variety of toxicants. Broadly, prenatal exposure to environmental toxins has been shown to impair memory and cause cell loss in the hippocampus (Karlsson et al., 2011, 2015). Heavy metal exposure decreases recognition memory and dendritic spine density which may lead to increased cell death (López et al., 2009; Pulido et al., 2019). Early exposure to the PAH BaP impairs memory and reduces CA1 dendrites (Das et al., 2019). Prenatal BPA exposure decreases CA1 spine synapses, although the same effect was not seen when BPA was administered during the juvenile period suggesting an age-dependent effect (Elsworth et al., 2013). Chronic mercury exposure induces TNFα protein expression in the hippocampus, which could lead to apoptotic events, a result only observed in male subjects (Curtis et al., 2011). Prenatal toxicant exposure *via* dredge-amended corn consumption could induce increased cell death that could manifest in a reduction in hippocampal volume.

These sex-specific effects, including those found in the present study, may be due to a neuroprotective mechanism facilitated by estrogen (Yao et al., 2011). Hippocampal cells are notably affected by estrogen across the lifespan. For example, CA1 dendritic spine and synapse density show a positive correlational relationship with estradiol administration in ovariectomized rats, estrogen promotes synaptic plasticity, and estrogen promotes neurite outgrowth *in vitro* cultures (*Reviewed in* Brann et al., 2007). There are conflicting findings in the research investigating estrogen as a neuroprotective mechanism. Although the importance of gonadal steroid hormones cannot be overlooked, there is another mechanism that may influence sex-specific brain and behavioral differences. Research suggests that thyroid hormone could also contribute to sex-specific differences. There are sex-specific peaks in thyroid hormone and thyroid hormone has been shown to contribute to cell neurogenesis during development, cell migration, and adult neurogenesis (Bernal, 2007; Remaud et al., 2014; reviewed in Baksi and Pradhan, 2021). Prenatal PCB exposure may interfere with thyroid hormone signaling in progeny (Gauger et al., 2004), which could lead to sex-specific differences in neural development and behavior later in life. While broad mixtures of toxicants may affect a number of neuroendocrine and developmental facets, with this preliminary study we are hesitant to suggest a mechanism driving the behavioral and neuroanatomical affects documented here. Future work will incorporate the full toxicant profile of dredge-amended and commercial feed corn into the proposed mechanism by which corn supplements affect neural development.

This study’s preliminary nature demonstrates the need for more research into implications of using dredged sediment as a soil amendment. Future studies will employ multiple doses of corn supplementation to determine a potential dose-response curve including corn that has been approved for human consumption, as commercially available feed corn may also contain COC. Additionally, a comparison will be made to animals without corn supplementation. A thorough battery of behavioral tests that assess multiple forms of cognition, including spatial learning/memory will be utilized. Future studies should also seek to determine the mechanisms by which dredge-amended and commercial feed corn alter the brain at the cellular level, assessing regional cell number and markers for apoptosis. This work should be pursued, as COC can magnify as they move up the food chain. If deviations from typical behavioral and neural development are being observed in a primary consumer, those same effects could be magnified in higher trophic levels due to increased exposure *via* bioaccumulation. Determining the potential effects of such soil amendments could have important clinical implications for animals and humans.

## Data availability statement

The raw data supporting the conclusions of this article will be made available by the authors, without undue reservation.

## Ethics statement

This animal study was reviewed and approved by the Bowling Green State University Institutional Animal Care and Use Committee (IACUC).

## Author contributions

JW, MR, and LS: conceptualization and methodology. KF, MC, and VR: investigation. KF and JW: data curation and writing. JW and LS: funding acquisition. All authors contributed to the article and approved the submitted version.
